# Improving the diagnosis of renal tumours of young people through integrated molecular analysis

**DOI:** 10.1007/s00432-026-06455-3

**Published:** 2026-04-03

**Authors:** Sarah M. Leiter, Aisosa O. Guobadia, Ben Fleming, Thankamma V. Ajithkumar, James N. Armitage, G. A. Amos Burke, Charlotte M. Burns, Nicholas Coleman, Helen Hatcher, Gail Horan, Anna-May Long, Sarah McDonald, Thomas J. Mitchell, James C. Nicholson, Thomas Roberts, Grant D. Stewart, John A. Tadross, Patrick S. Tarpey, Claire Trayers, Jamie Trotman, James A. Watkins, Anne Y. Warren, Gordan M. Vujanic, Ruth Armstrong, Sam Behjati, C. Elizabeth Hook, Matthew J. Murray

**Affiliations:** 1 Department of Paediatric Haematology and Oncology, Cambridge University Hospitals NHS Foundation Trust, Cambridge, CB2 0QQ UK; 2 Department of Paediatrics, School of Clinical Medicine, University of Cambridge, Cambridge, CB2 0SP UK; 3 Department of Radiology, Cambridge University Hospitals NHS Foundation Trust, Cambridge, CB2 0QQ UK; 4 Department of Clinical Oncology, Cambridge University Hospitals NHS Foundation Trust, Cambridge, CB2 0QQ UK; 5 Department of Urology, Cambridge University Hospitals NHS Foundation Trust, Cambridge, CB2 0QQ UK; 6 Cancer Research UK Clinical Trials Unit, School of Medical Sciences, University of Birmingham, Birmingham, B15 2TT UK; 7 Department of Paediatric Oncology, Birmingham Women’s and Children’s NHS Foundation Trust, Birmingham, B15 2TG UK; 8 Department of Histopathology, Cambridge University Hospitals NHS Foundation Trust, Cambridge, CB2 0QQ UK; 9 Department of Pathology, University of Cambridge, Cambridge, CB2 1QP UK; 10 Department of Oncology, Cambridge University Hospital NHS Trust, Cambridge, CB2 0QQ UK; 11 Department of Paediatric Surgery, Cambridge University Hospitals NHS Foundation Trust, Cambridge, CB2 0QQ UK; 12 Department of Surgery, School of Clinical Medicine, University of Cambridge, Cambridge, CB2 0SP UK; 13 East Genomics Laboratory Hub (E-GLH) Genetics Laboratory, Cambridge University Hospitals NHS Foundation Trust, Cambridge, CB2 0QQ UK; 14 CRUK Cambridge Centre, University of Cambridge, Cambridge, CB2 0QQ UK; 15 MRC Metabolic Diseases Unit, Wellcome Trust-Medical Research Council Institute of Metabolic Science, University of Cambridge, Cambridge, CB2 0QQ UK; 16 Department of Pathology, Sidra Medicine, Doha, Qatar; 17 Department of Pathology and Laboratory Medicine, Weill Cornell Medicine - Qatar, Doha, Qatar; 18 Department of Clinical Genetics, Cambridge University Hospitals NHS Foundation Trust, Cambridge, CB2 0QQ UK; 19 Wellcome Sanger Institute, Hinxton, Cambridge, CB10 1SA UK

**Keywords:** Wilms tumour, Renal cell carcinoma, Whole genome sequencing, Cancer predisposition, Molecular analysis

## Abstract

**Background:**

Renal tumours account for one in twenty paediatric cancers, with Wilms tumour (WT) the most common in young children and renal cell carcinoma (RCC) predominating in adolescents and young adults. Diagnostic work-up has traditionally focused on clinical features, radiology, and histology, with a limited role for molecular analysis. However, it is estimated that up to one-third of children with WT have underlying cancer predisposition, which could necessitate prolonged treatment and intensive follow-up.

**Methods:**

Here we describe five children and young adults treated at a single regional centre in England who had paired tumour and germline whole genome sequencing (WGS) as part of their routine diagnostic work-up.

**Results:**

One child diagnosed radiologically with a WT underwent pre-operative chemotherapy with good clinical and imaging response. Histological examination of the resection raised concerns over RCC; however, WGS was able to confirm that this was a WT with pathognomonic somatic WT changes. A young adult with upfront nephrectomy had a difficult-to-classify tumour; WGS revealed a novel *ERC1-CCNY* fusion as a likely novel driver event. Two further children, who did not meet clinical criteria for cancer predisposition testing, had predisposition syndromes identified via agnostic WGS. Finally, a child with piebaldism had a WT-associated *REST* deletion identified early through critical clinical thinking and expedited microarray.

**Conclusion:**

We highlight that molecular analysis, particularly agnostic WGS, has a key routine role in the care of children with renal tumours. It is likely that outcomes for these young people have been improved through more accurate diagnosis and early detection of cancer predisposition.

**Supplementary Information:**

The online version contains supplementary material available at 10.1007/s00432-026-06455-3.

## Introduction

Renal cancers account for approximately 5% of all childhood tumours, equating to around 1000 new cases in Europe per year (Pastore et al. 2006; Nakata et al. 2020). Wilms tumours (WT) account for ~ 90% of these (Pastore et al. 2006; Nakata et al. 2020), with overall survival (OS) of 90% and 75% for those with localised and metastatic disease, respectively (Brok et al. 2018; Malogolowkin et al. 2013). Historically, an estimated 10% of patients with WT were thought to have an underlying cancer predisposition syndrome. More recently, this has been projected to be as high as one in three (Hol et al. 2022), particularly in the presence of multiple nephrogenic rests, which are known precursor lesions for WT. Screening for predisposition syndromes has traditionally been limited to those children with nephrogenic rests and/or nephroblastomatosis (multifocal involvement of the kidneys with nephrogenic rests) and specific clinical features including young age of presentation, bilateral or multi-focal tumours, and hemihypertrophy/macrocephaly (Hol et al. 2022). The prompt identification of children with underlying cancer predisposition is important as they should be considered for nephron-sparing surgery (NSS). These patients also require longer surveillance to detect development of further renal and/or extrarenal tumours and, where appropriate receive a longer duration of chemotherapy (Kim et al. 2021; Hol et al. 2021a).

Paediatric non-WT (NWT) are a heterogeneous group, rare, can be challenging to diagnose, and the neoplasms vary in malignant potential, with concomitant variation in long-term clinical outcomes (Nakata et al. 2020). Despite their heterogenicity, there is sometimes an overlap in histologic patterns and cell types with WT compounding diagnostic dilemma (Ooms et al. 2020). For example, papillary-type renal cell carcinoma (pRCC) and metanephric adenomas are differential diagnoses for epithelial WT due to their overlapping histological morphology (Ooms et al. 2020). Paediatric RCC make up ~ 2–6% of childhood renal tumours; with prevalence increasing with age, from 3.5% in those 0–14 years (y) to 50–70% at 14–19 y (Beck et al. 2022; Beek et al. 2021). Of note, RCCs are typically poorly chemotherapy and radiotherapy sensitive and thus require early radical nephrectomy, with 5-year OS for paediatric metastatic RCC reported in one series to be only 15% (Beek et al. 2021). Given such differences in treatment and outcomes, accurate and timely diagnosis of renal tumours is essential. As our understanding of cancer biology evolves, the role of genomics in diagnosis and treatment is increasingly being recognised (Newman et al. 2021; Villani et al. 2022).

To evaluate the current use of molecular diagnostics within paediatric renal tumours: we reviewed the two commonly used clinical treatment guidelines for paediatric renal tumours, The Children’s Oncology Group (COG) guideline from North America (Balis et al. 2021), and the International Society of Paediatric Oncology Renal Tumour Study Group (SIOP-RTSG) Guideline (Vujanić et al. 2018) used in Europe, Asia and Southern America.

The COG guideline advocates upfront nephrectomy in most circumstances, whilst under SIOP-RTSG children receive pre-operative chemotherapy to allow for the overall downstaging of tumours (Vujanić et al. 2018). Furthermore, in Europe, children who are presumed to have WT based on typical age, biochemistry, and imaging, receive chemotherapy without biopsy (Pater et al. 2021). In both guidelines, epidemiological, radiological, and histological features are currently used to define tumour type, with the mainstay of definitive diagnosis being histological examination (Ooms et al. 2020; Sebire and Vujanic 2009), with focused immunohistochemistry (IHC) panels based on initial histology review.

Regarding molecular investigations, neither guideline advocates for routine somatic molecular testing for diagnosis of WT. The SIOP-RTSG recommends considering molecular profiling at relapse for consideration of potential clinical trials. Furthermore, the SIOP-RTSG guideline makes reference to molecular testing in NWT depending on the histopathological diagnosis e.g. *TFE3* or *TFEB* translocations in RCC. With regard to risk stratification, the COG guideline uses loss-of-heterozygosity (LOH) of 1p and 16q for risk stratification whilst no molecular risk stratification is present in the current SIOP-RTSG guideline. Germline cancer predisposition syndrome (CPS) testing is recommended by the COG for children with examination findings consistent with a predisposition syndrome including aniridia and hemihypertrophy. The SIOP-RTSG guideline makes no direct recommendations for CPS testing in WT but does have a treatment guideline for children presenting with a likely underlying CPS and/or bilateral tumours. For these patients, NSS and a prolonged adjuvant chemotherapy phase is recommended.

Paired tumour and germline whole genome sequencing (WGS) has proven extremely useful in cancer management (Trotman et al. 2022; Jobanputra et al. 2022; Hodder et al. 2024), and has been routinely adopted by some paediatric and young adult cancer services in England, including our centre.

Here, we describe a series of challenging renal tumour cases with non-characteristic histological appearances, as well as cases with unexpected cancer predisposition. Molecular analysis including paired tumour and germline WGS, together with careful interpretation of clinical, radiological, and histological appearances helped to inform final integrated diagnoses and management, with likely influence on patient outcomes. The series described here supports our advocacy for agnostic molecular studies for all young patients presenting with renal tumours. Such findings have important implications for future pathological classifications, which will continue to evolve as our understanding of these rare tumour groups improve.

## Subjects and methods

All young people presented to a single regional principal treatment centre for children (< 16 y) with cancer and a Teenage/Young Adult oncology service (16–24 y). The paediatric patients were diagnosed between February 2022 and June 2023; during this period a total of thirteen children with kidney cancers were diagnosed and had clinical paired tumour/germline WGS as a standard part of their clinical care. Very brief summary details of three patients (cases 1, 3 and 5) are included in a larger published dataset (*n* = 281) demonstrating the overarching benefit of WGS in children with suspected cancer (Hodder et al. 2024). All molecular testing, including WGS, was undertaken in a United Kingdom Accreditation Service (UKAS)-accredited laboratory to standard ISO 15189, and as previously described (Hodder et al. 2024). Consent was obtained from patients/families for review of clinical records including pathology, radiology, molecular results, collection of relevant clinical details, and publication in anonymised form (Table 1).

**Table 1:** Summary table describing clinical, radiological, histological, and molecular features of all patients with final clinical diagnosis and management

CaseID	Age at diagnosis (y)	Sex	Ethnicity	Probable radiological diagnosis	Upfront biopsy performed?	Histological differential diagnosis	IHC markers	Histological uncertainty?	Molecular assays performed	Molecular results (summary)	Final integrated diagnosis	Final treatment strategy (UMBRELLA)
1	3	Male	White	Metastatic WT	No—chemotherapy first	Epithelial WT vs RCC	INI-1, Vimentin, Cytokeratin + ve | CK7 focally + ve | WT1, CD10 mixed	Yes	FISH | pWGS	*FBXW7* | 1p loss | 1q gain	Sporadic stage III WT	Stage III WT
2	19	Female	White	Localised heterogenous renal mass	No—upfront nephrectomy	Epithelial WT vs RCC	WT1, Vimentin, PAX8, CD56, WT1, Glypican-3, MNF116, Cyclin-D1, INI-1 + ve | BRAF, ALK, AMACR, CD57, EMA, CK7, AE1/3, CA-IX, TTF1, S100, Desmin, Melan-A -ve	Yes	FISH | pWGS | RNA Fusion panel	Chromosomes 10, 11, 12 rearrangement | *PDGFD* | *ERC1::CCNY* | *PIK3C2G*	RCC NOS	Nephrectomy alone
3	0.6	Male	White	Localised solid renal mass	Yes	Epithelial WT with nephrogenic rests	INI-1, Pan-keratin, CD56, WT1 + ve | Racemace focally + ve	No	pWGS	*TRIM28* mosaicism	Syndromic stage I WT	Syndromic stage I WT
4	8	Male	White	Localised WT	No—upfront nephrectomy	Triphasic WT	Not required as haematoxylin & eosin (H&E) staining diagnostic	No	pWGS	*DIS13L2* (germline) | *AMER1* | *CREBBP*	Syndromic stage I WT	Syndromic stage I WT
5	1	Male	Mixed	Renal mass with extrarenal component	Yes	Triphasic WT	INI-1 + ve | WT1, PAX8, CD56, p53 patchy + ve	No	Germline microarray | pWGS	4q deletion encompassing *KIT & REST* (germline) | *REST* | 11p LOH	Syndromic stage II WT	Syndromic stage II WT

CCSK, clear cell sarcoma of kidney; CNV, copy number variant; FISH, fluorescent in situ hybridisation; IHC, immunohistochemistry; LOH, loss of heterozygosity; NGS, next generation sequencing; NOS, not otherwise specified; pRCC, papillary renal cell carcinoma; pWGS, paired whole genome sequencing; RCC, renal cell carcinoma; WT, Wilms tumour; y, years, + ve, positive, − ve, negative

## Results

### Case 1

**Fig. 1:**
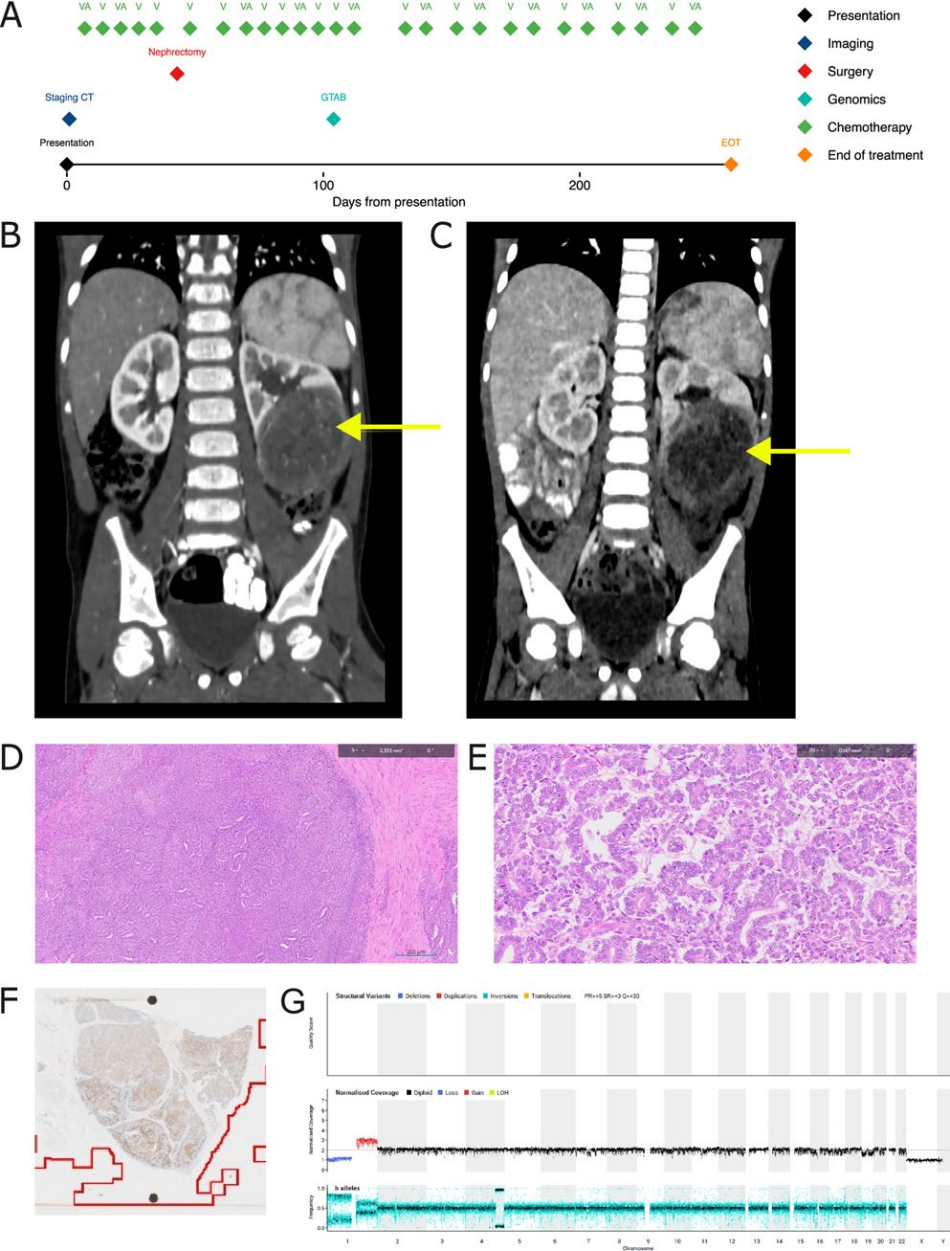
Case 1 clinical summary demonstrating response to chemotherapy and representative investigations. **A** Timeline of clinical events. Of note that only radiological investigations referenced in the manuscript are included in this figure. **B** Coronal reformat from a corticomedullary phase CT of the abdomen and pelvis, performed at presentation, showing a large, heterogeneous left lower pole renal mass (arrow). **C** Equivalent coronal reformat from a follow-up CT performed one month later, showing increased tumour necrosis consistent with treatment response but no substantial decrease in overall size (arrow). **D**, **E** H&E-stained nephrectomy slices showing epithelial differentiation and papillary-type morphology, respectively. **F** WT1 staining, showing positive staining in areas of morphological epithelial differentiation interspersed with negative areas. **G** WGS-derived CNV plot showing chromosome 1p loss and 1q gain. A, Actinomycin D; CNV, copy number variants; CT, computerised tomography; EOT, End of Treatment; GTAB, genomic tumour advisory board; H&E, haematoxylin and eosin; V, Vincristine, WGS, whole genome sequencing

Timeline: Fig. 1A. A 3-year-old White male presented with frank haematuria and palpable left flank mass, confirmed by imaging (Fig. 1B); CT also showed left para-aortic lymphadenopathy. No other metastases were identified, and serum lactate dehydrogenase (LDH) was normal. Clinical and radiological findings were consistent with WT and, as per UMBRELLA guidelines (Vujanić et al. 2018), pre-operative ‘VA’ (vincristine-actinomycin) chemotherapy was delivered. Reassessment imaging showed stable tumour size but with evidence of treatment response (Fig. 1C). Following open radical left nephrectomy, histology showed epithelial differentiation (Fig. 1D) and papillary-type morphology (Fig. 1E) with psammomatous calcification, suggestive of (*TFE3/TFEB*) translocation-type RCC. There was no evidence of anaplasia. In addition, no definite blastema or stromal components were identified, making WT unlikely. IHC for WT1 showed a highly unusual staining pattern within the areas of morphological epithelial differentiation, with positive areas interspersed with completely negative areas (Fig. 1F), not typically observed in WT. INI-1 staining was retained, excluding malignant rhabdoid tumour. Further IHC was non-contributory. Interphase FISH for the *TFE3* translocation was inconclusive and no *TFE3/TFEB* IHC was available. Sampled abdominal lymph nodes showed tumour involvement. Favoured local (epithelial WT) and external (RCC) pathology opinions were divergent. WGS performed as per local clinical practice detected a somatic *FBXW7* missense variant (p.Arg505Cys, Supplementary Table S1), chromosome 1p loss and 1q gain (Fig. 1G), consistent with epithelial WT (Williams et al. 2010; Treger et al. 2019). Accordingly, as per UMBRELLA guidelines for intermediate risk, stage III disease (nodal involvement) (Vujanić et al. 2018), he was treated with left flank radiotherapy (14.4 Gy) and six months ‘VA’ chemotherapy. The patient remains in complete remission (CR) 28 months following end-of-treatment (EOT).

### Case 2

**Fig. 2:**
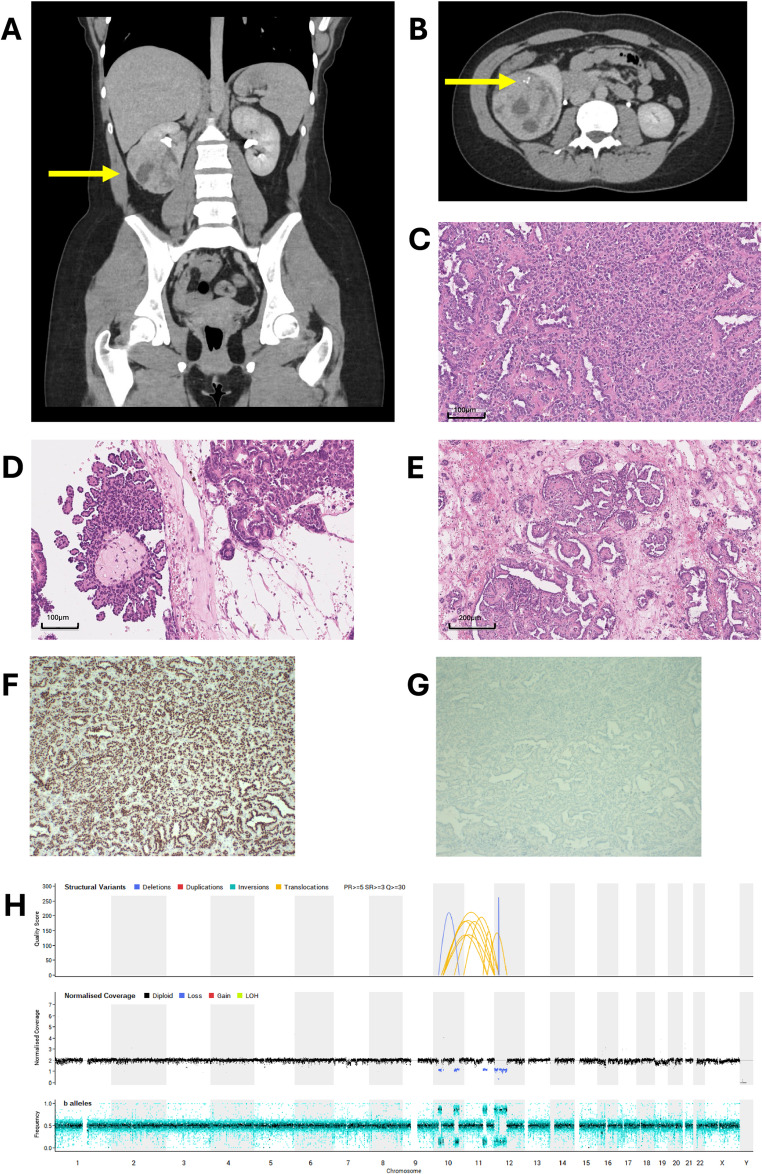
Case 2 clinical summary demonstrating histological ambiguity of the renal tumour. **A**, **B** Coronal and axial reformats, respectively, from an early urographic phase CT of the abdomen and pelvis performed at presentation, showing a rounded, heterogeneous right interpolar renal mass (arrows). Tiny internal foci of calcification were demonstrated on the axial view. **C**–**E** H&E-stained nephrectomy specimen showing areas of tumour with, **C** a sheet-like solid arrangement (centre) of cells and angulated tubules, **D** papillary structures (left) and compact tubules, **E** loosely arranged glomeruloid structures (centre) and tubules within more abundant fibrous stroma. **F** Positive nuclear WT1 staining. **G** Negative PAX8 staining. H) WGS-derived CNV plot showing a complex rearrangement of chromosomes 10, 11, and 12. CNV, copy number variants; CT, computerised tomography; EOT, End of Treatment; GTAB, genomic tumour advisory board; H&E, haematoxylin and eosin; WGS, whole genome sequencing

A 19-year-old White female presented with an incidental ultrasound finding of a right-sided renal mass, confirmed by CT (Fig. 2A, B), with no evidence of metastasis. Following upfront laparoscopic right radical nephrectomy, standard in adult oncological practice, histology revealed a predominantly solid, well-circumscribed tumour surrounded by a thin fibrous capsule with variably sized cystic areas. Based on morphology (Fig. 2C–E), differential diagnoses included metanephric adenoma, epithelial WT, and pRCC. WT1 staining was positive (Fig. 2F) in keeping with metanephric adenoma or WT, likely excluding pRCC. The IHC profile was not consistent with metanephric adenoma however, due to the absence of BRAF staining (Fig. 2G) and the presence of some mitotic activity. Furthermore, despite WT1 staining, overall features were not typical for WT due to the prominent papillary morphology. Finally, an ALK-rearranged RCC was excluded through negative ALK IHC and interphase FISH. Following discussion with The Children and Young People’s Cancer Association (CCLG) National Renal Advisory Panel (2024), the consensus was that the radiological and histological appearances were most consistent with an epithelial WT; accordingly, treatment with WT-based chemotherapy and flank radiotherapy was recommended. In contrast, international pathology experts reported the tumour as a rare type of RCC and unlikely WT. WGS revealed a complex rearrangement involving chromosomes 10, 11, and 12 (Fig. 2H). This included heterozygous loss of *PDGFD* on chromosome 11 and rearrangement of the remaining copy, preserving the tyrosine kinase inhibitor domain; a rearrangement at this locus (albeit with a different partner) has been previously described in WT (Ma et al. 2018). A complex rearrangement involving the *ERC1* locus, deletion in the region encompassing *PIK3C2G*, and a rearrangement in *CCNY* was also observed (Supplementary Table S1). No typical genomic signatures of WT, papillary RCC, or metanephric adenoma were identified. RNA sequencing was used to interrogate the WGS findings further and confirmed a novel *ERC1-CCNY* fusion (Supplementary Table 2). The *ERC1-CCNY* fusion was in frame and juxtaposed the entire *ERC1* gene to the 3′ portion of *CCNY* from exon 8, which included a portion of the cyclin domain (and the interaction binding sites), whilst removing some of the 5′ regulatory sites. A rearrangement of *CCNY*, with *CCNY* as the 5′ partner at the same breakpoint but with a different 3′ partner (*CCDC82*), has been described in WT (Ma et al. 2018). A further round of international pathology review was instituted in view of the discovery of this novel fusion, following which the tumour was designated as an RCC ‘not otherwise specified’ (NOS), non-clear cell, non-*ALK*-rearranged subtype with an *ERC1-CCNY* fusion. Given complete resection, no evidence of metastatic disease, and low mitotic count, no further chemotherapy was felt to be indicated, and surveillance was commenced. The patient is now 33 months post-surgery with no recurrence.

### Case 3

**Fig. 3:**
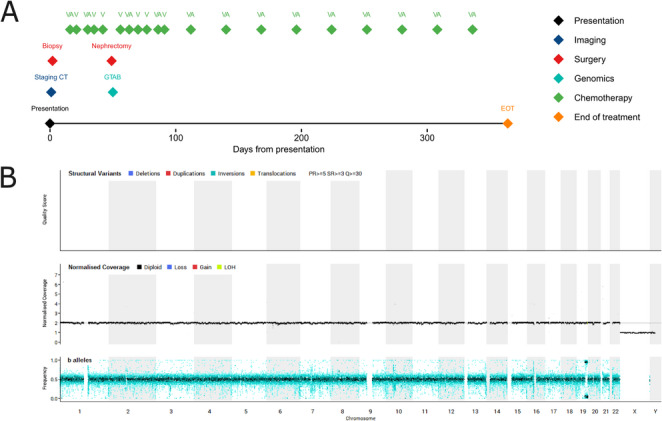
Case 3 clinical summary of events and representative investigations. **A** Timeline of clinical events. Note that only radiological investigations referenced in the manuscript are included in this figure. **B** WGS-derived CNV plot for showing ‘flat’ landscape with no chromosomal gains or losses but chromosome 19 LOH. A, Actinomycin D; CNV, copy number variants; CT, computerised tomography, EOT, End of Treatment; GTAB, genomic tumour advisory board; H&E, haematoxylin and eosin; LOH, Loss of Heterozygosity; V, Vincristine; WGS, whole genome sequencing

Timeline: Fig. 3A. A 7-month-old White male presented with a right-sided asymptomatic abdominal mass with normal urinary catecholamines and serum LDH. No overgrowth nor syndromic features were present, and he did not qualify for predisposition testing as per the Genomics England Test Directory at the time of his diagnosis (NHS England 2019). A CT scan showed a well-defined solid mass without evidence of metastasis or contralateral renal tumours (Supplementary Fig. 1A). In view of his age (< 1 year) and the absence of metastases, he underwent a biopsy, confirming WT on a background of nephrogenic rests (Supplementary Fig. 1B). Only epithelial elements were identified within the tumour, and as such this was subclassified as an epithelial type WT. He was started on WT treatment as per UMBRELLA guidelines (Vujanić et al. 2018) and pre-operative imaging showed substantial reduction in tumour size (Supplementary Fig. 1C). Open nephrectomy confirmed intermediate risk epithelial-type WT arising from large nephrogenic rests without lymph node involvement (stage I). Following right radical nephrectomy, he commenced post-operative ‘VA’ chemotherapy. WGS revealed a somatic *TRIM28* truncating variant (Supplementary Table S1) on the background of loss-of-heterozygosity (LOH) of the same locus at chromosome 19 without other chromosomal alterations (Fig. 3B). The *TRIM28* variant was confirmed in the tumour with a variant allele frequency (VAF) of 0.85, in normal kidney with a VAF of 0.0035 (one sample tested), but was undetectable in lymphocyte DNA, confirming a mosaic *TRIM28* variant with variable expression in body tissues. Germline and mosaic *TRIM28* variants have previously been described in association with familial WT (Wegert et al. 2024; Mahamdallie et al. 2019), and the child was treated as per syndromic WT guidelines with monthly ‘VA’ chemotherapy for one year following surgery. He is now 34 months post-EOT without evidence of recurrence.

### Case 4

**Fig. 4:**
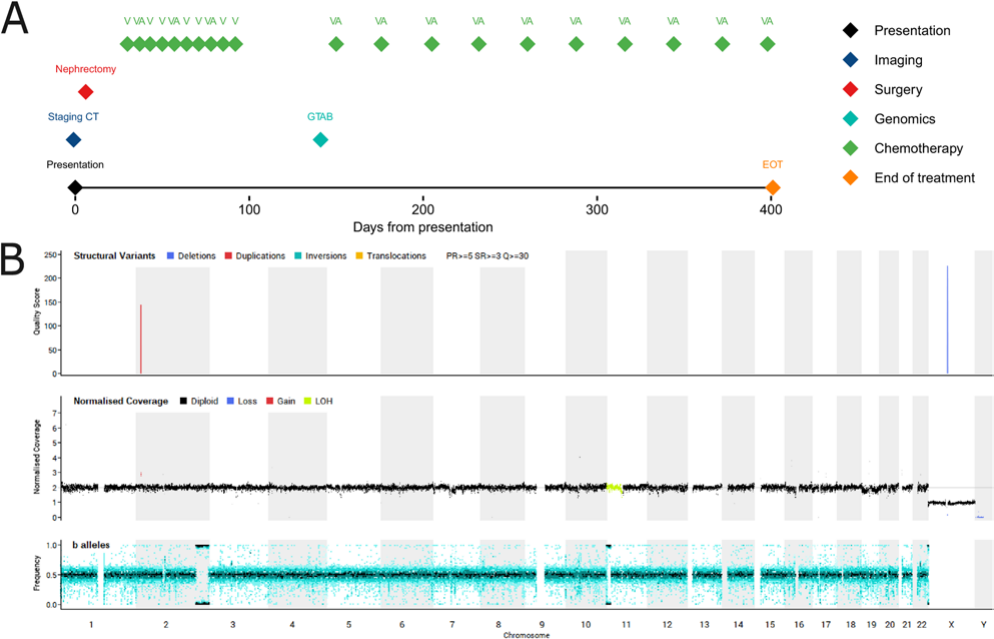
Case 4 clinical summary of events and representative investigations. **A** Timeline of clinical events. Note that only radiological investigations referenced in the manuscript are included in this figure. **B** WGS-derived CNV plot demonstrating 11p LOH. A, Actinomycin D; CNV, copy number variants; CT, computerised tomography; EOT, End of Treatment; H&E, haematoxylin and eosin; GTAB, genomic tumour advisory board; LOH, Loss of Heterozygosity; V, Vincristine; WGS, whole genome sequencing

Timeline: Fig. 4A. An 8-year-old White male presented with abdominal pain following minor trauma. No overgrowth nor syndromic features were present, nor was there a family history of cancer. An ultrasound and CT scan showed a left-sided renal mass with claw sign, consistent with WT (Supplementary Fig. 2A). No local or distant metastases were identified. In view of potential radiological concern regarding tumour rupture however, he underwent an upfront open nephrectomy. As per the UMBRELLA guidelines for chemotherapy-naïve tumours, histology confirmed a non-anaplastic triphasic WT (Supplementary Fig. 2B, C), intermediate-risk, stage I, with no actual evidence of any capsular breach/rupture. There was no evidence of nephrogenic rests nor anaplasia on the resection specimen. The patient therefore only received a short course of post-operative chemotherapy as per the UMBRELLA guidelines (Vujanić et al. 2018). EOT MRI showed no evidence of disease. Shortly after EOT, WGS identified a heterozygous germline *DIS3L2* variant, consistent with a germline WT predisposition syndrome (Friedman et al. 2023; Astuti et al. 2012; Peer et al. 2025) (Fig. 4B, Supplementary Table S1) as well as somatic *AMER1* and *CREBBP* variants. Accordingly, after discussion with the family, chemotherapy was recommenced as per syndromic WT guidelines (monthly ‘VA’ post-operative chemotherapy for one-year post-surgery) and the patient remains in CR 16 months after the EOT. Genetic counselling was offered to the family and cascade testing showed that the *DIS3L2* variant was inherited maternally.

### Case 5

**Fig. 5:**
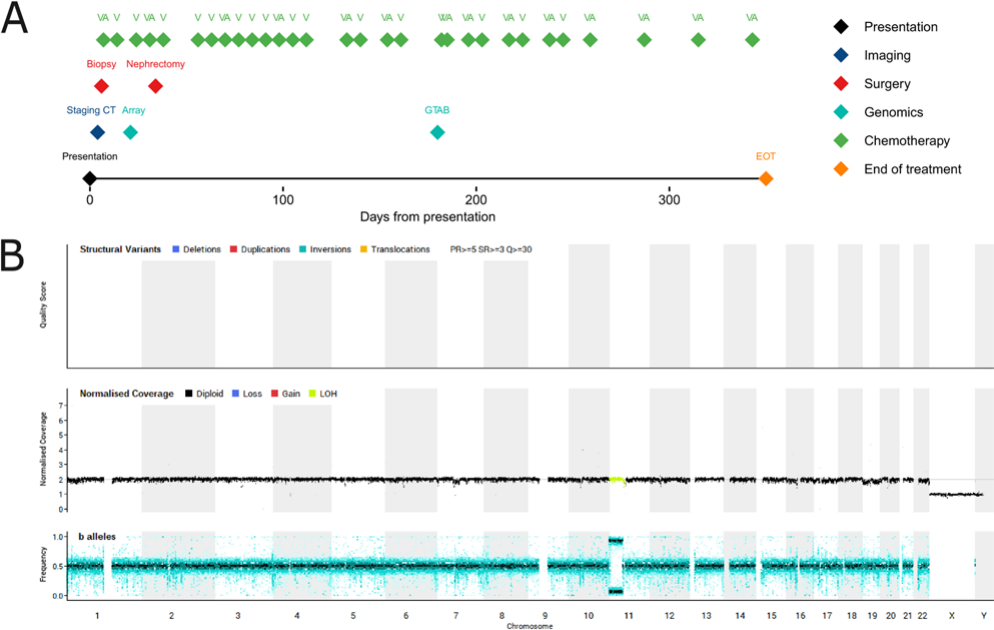
Case 5 clinical summary of events and representative investigations. **A** Timeline of clinical events. Note that only radiological investigations referenced in the manuscript are included in this figure. **B** WGS-derived CNV plot showing 11p LOH; it should be noted that the germline 4q deletion is too small to be visualised on this landscape plot. A, Actinomycin D; CNV, copy number variants; CT, computerised tomography; EOT, End of Treatment; GTAB, genomic tumour advisory board; H&E, haematoxylin and eosin; LOH, Loss of Heterozygosity; V, Vincristine; WGS, whole genome sequencing; WT, Wilms Tumour

Timeline: Fig. 5A. An 18-month-old male of Mixed ethnicity presented with a few weeks’ history of abdominal symptoms. An ultrasound showed a right upper quadrant mass. CT imaging showed a right-sided renal tumour with extensive extrarenal components, not typical for WT (Supplementary Fig. 3A). Urinary catecholamines and serum LDH were normal, and biopsy showed a triphasic WT with some areas of glomerular sclerosis. He did not qualify for predisposition testing as per the Genomics England Test Directory at the time of diagnosis (NHS England 2019). Following standard pre-operative ‘VA’ chemotherapy induction, imaging showed interval tumour size reduction (Supplementary Fig. 3B). Of note, both the child and his mother had piebaldism, commonly caused by *KIT* deletions on chromosome 4q (Ezoe et al. 1995). Indeed, the mother had previously had genetic testing and a microarray confirmed a heterozygous chromosome 4q deletion [4q12(53145311_56655159)]. Further interrogation of the 4q genomic region by his treating clinician identified that *KIT* was located ~ 2 megabases (2 Mb) upstream of the WT predisposition gene *REST* (Mahamdallie et al. 2015). Accordingly, following the start of chemotherapy, an urgent germline microarray was performed and revealed an expanded 4q deletion encompassing both *KIT* and *REST*. NSS was not feasible in view of radiological concerns over tumour rupture, and he thus underwent a right open nephrectomy, with histology confirming an intermediate-risk stage II WT without evidence of anaplasia or nephrogenic rests (Supplementary Fig. 3C). The child was treated as per syndromic WT for one-year post-surgery as per the UMBRELLA guidelines (Vujanić et al. 2018). WGS confirmed the germline 4q deletion and revealed a somatic truncating *REST* variant as well as 11p LOH (Fig. 5B, Supplementary Table S1). There is no evidence of recurrence 27 months post-EOT.

## Discussion

With increased clinical implementation of WGS, the timely and positive impact on assisting diagnosis, subclassification, and prognostication for patients with cancer has been highlighted (Villani et al. 2022; Hodder et al. 2024; Wadensten et al. 2023; Ryan et al. 2023). In this series of paediatric and young adult cases, summarised in Table 1, encompassing both typically ‘paediatric-type’ (e.g., WT) and ‘adult-type’ (e.g., RCC) tumours, we have demonstrated how agnostic molecular analysis, alongside clinical, radiological, and histological information, facilitates the delivery of fully integrated diagnoses allowing timely and optimal management, including the potential identification of novel entities.

### The role of somatic molecular testing in young people with difficult-to-classify renal tumours

The first two cases demonstrate the value of molecular analysis when histological uncertainties exist, the merit of which extends even to small biopsies. Renal tumours showing overlapping or mixed histological features present a diagnostic challenge (Ooms et al. 2020; Pfister et al. 2022), which without further genomic testing, may be misclassified or remain unclassified. Clinical utilisation of WGS has been shown to provide invaluable diagnostic information in cases of uncertainty, changing working diagnoses or refining diagnoses into clinically distinct subtypes (Trotman et al. 2022; Wadensten et al. 2023; Ryan et al. 2023). For Case 1, substantial uncertainty over the initial diagnosis (which had been based on radiology and epidemiology) was raised by histopathological appearances. WGS confirmed WT through both the copy number profile as well as the somatic *FBXW7* variant and enabled continuation of the planned cytotoxic treatment.

In the second case, WGS did not show any genomic features typical of WT or RCC, but instead rearrangements of chromosomes 10, 11, and 12. WGS was able to elucidate a probable novel driver fusion in *ERC1-CCNY*; which was confirmed to be translated in RNA studies. These two fusion partners have not previously been reported, although a *CCNY* fusion with the same break-point has been shown in WT (Ma et al. 2018). Overall, the patients age, presentation, histology, and absence of WT-typical genomic changes led to a classification as RCC NOS. This provided valuable information to both the clinical team and the family. Without molecular analysis, both cases would have remained without a formal consensus diagnosis, and/or unclassified, with the potential to adversely impact upon management decisions and thus outcome. Consistent with the RCC NOS consensus diagnosis, the patient remains well 33 months post-EOT with surgery alone, without recourse to any systemic chemotherapy.

### Identification of cancer predisposition in young people with renal tumours

The three latter cases demonstrate the value of both maintaining a high index of clinical suspicion and undertaking appropriate molecular analyses in establishing which children may require modified WT treatment in view of an underlying predisposition (Vujanić et al. 2018). It is estimated that 8–18% of children with cancer have an underlying genetic predisposition (Kim et al. 2021; Zhang et al. 2015; Akhavanfard et al. 2020) and that it is not possible to identify all cases based on the history and radiological features alone (Byrjalsen et al. 2020). The Generation Study (2024) in England seeks to identify, among others, children with underlying WT predisposition, aiming to provide further insight into the childhood and life-time risk of developing such malignancies. For children with WT predisposition, who may develop second renal tumours, NSS should be considered to reduce the longer-term risk for renal replacement therapy (Vanden Berg et al. 2016).

None of the children reported would have met the criteria for the nationally defined English ‘R220 Wilms tumour with features suggestive of predisposition’ panel as set out by the Genomic Test Directory at the time of diagnosis (NHS England 2019), although this has since been expanded to include all children less than two years old. Even if clinicians had requested the panel, the genomic variants identified by paired WGS for Cases 3 would not have been detected. With current TAT of approximately four months for some cancer predisposition panels, paired WGS therefore offers a faster, broader approach which facilitates manual data review and the evolution of testing algorithms.

Adaptability of the clinical testing algorithm is important as our knowledge of WT predisposition is rapidly evolving. Mosaicism is being recognised more frequently in children with WT—both at an early embryonic phase as well as a renal-specific phenomenon (Wegert et al. 2025; Treger et al. 2025). Indeed, it has been suggested that sampling multiple parts of the ‘normal’ background kidney should be undertaken to evaluate if a driver variant is secondary to renal mosaicism (Wegert et al. 2024; Diets et al. 2019; Hol et al. 2021b). In Case 3, the somatic *TRIM28* variant has indeed been confirmed in surrounding kidney, resulting in altered management. The heterozygous *DIS3L2* exon 9 deletion identified in Case 4 has recently been reported in multiple WT cases (Friedman et al. 2023; Peer et al. 2025). Interestingly, in the largest case series to date, van Peer et al. described 34 children with this mutation of whom a significant proportion had high-risk, bilateral, or metastatic disease (Peer et al. 2025). Our patient had intermediate risk, stage I tumour. Early coincidental WT detection, following an injury, could be contributing to this observation in our case.

This is the first report to our knowledge of a child (Case 5) with piebaldism and a WT. Furthermore, such substantial variation in one of the breakpoints of a familial interstitial copy number loss is exceptionally rare and has not been observed in our laboratory before nor, to our knowledge, been reported in the published literature. The difference in size between the mother and child’s copy number loss could possibly be explained by unequal non-homologous crossover during meiosis, which would lead to a low risk to siblings of Case 5.

### WGS as a single assay for the evaluation of renal tumours in young people

With ever-evolving integration of molecular genomic analysis for the diagnosis, risk stratification, and treatment of children and adults with cancer, the question over the best single/combination of assays arises. WGS is a useful tool in this setting (Jobanputra et al. 2022; Hodder et al. 2024; Leung et al. 2024; Bagger et al. 2024), however the longer TAT in some centres can create challenges. The ability to evaluate copy number profiles as well as single nucleotide or small variants, to allow for germline subtraction, and the ability to perform an agnostic (re-)analysis, makes WGS the single most informative assay and, accordingly, preferable over a more limited panel. Furthermore, when ordering individual molecular assays, a major limitation is that the requesting clinician has to know what answers are expected, whilst agnostic WGS may provide unexpected but clinically relevant information. In our experience, using post-chemotherapy nephrectomy samples for WGS provided good quality data using standard pipelines in a UKAS-accredited laboratory.

It should be noted that purely ‘algorithm-based’ WGS, i.e., using WGS as a bioinformatic assay without clinical context, is unlikely to be able to capture and define the nuances observed in many clinical cases. A multidisciplinary Genomic Tumour Advisory Board (GTAB) approach including treating clinicians, pathologists, geneticists, and clinical scientists remains essential to define novel entities in the context of the full and broadest clinical picture.

### Limitations

Renal tumours in young people are a rare entity, and we acknowledge that our case series is of modest size. Routine implementation of WGS has previously been evaluated for its impact on children presenting with cancers but this is the first study, to our knowledge, looking specifically at WGS in renal tumours. Accordingly, larger studies focusing specifically on renal tumours in young people are required to determine the proportion of patients who benefit from WGS. It should be noted that the cost of WGS has fallen rapidly over the past decade; a formal economic cost–benefit analysis however is beyond the scope of this work. Balancing the cost of materials, time, and sequencing, with the potential for more accurate diagnosis and precision medicine is complex and will require prolonged follow-up to formally demonstrate improved patient care and cost-effectiveness.

## Conclusion

In summary, this case series demonstrates the critical role of agnostic molecular analysis to reach integrated diagnoses in renal tumours of young people, particularly beneficial given the histologically challenging cases described here. The series provide s early evidence of the direct benefit of using agnostic somatic and germline molecular testing, including WGS, as a standard of care for obtaining genomic information in all paediatric/young adult patients presenting with renal tumours. Over this period, five of thirteen paediatric patients who underwent WGS has significant molecular results challenging the current practice of not routinely performing molecular analysis in paediatric renal tumours. Genomic findings will need to be reflected in future iterations of the World Health Organisation (WHO) classification and will support clinicians in decision making to facilitate optimal patient outcomes.

## Supplementary Information

Below is the link to the electronic supplementary material.

Supplementary Material 1

## Data Availability

As per Genomics England whole genome sequencing consent, we are unable to share raw sequencing data for the patients described. Summary data is included in the publication. Researchers may apply to join the Genomics England Research Network to access anonymised raw sequencing data, variant calls, signature analysis, quality metrics, and a summary of findings submitted to Genomic Laboratory Hubs.
