# Supplementary material for: Improving the diagnosis of renal tumours of young people through integrated molecular analysis
Source: J Cancer Res Clin Oncol. 2026 Apr 3;152(4):81. doi: 10.1007/s00432-026-06455-3 (PMC13049143; doi:10.1007/s00432-026-06455-3)
Supplement: Supplementary file 1 — Supplementary Material 1 [file 432_2026_6455_MOESM1_ESM.pdf]

Improving the diagnosis of renal tumours of young people through integrated molecular analysis
Leiter SM et al

<u>Supplementary Tables & figures</u>

Supplementary Table 1 – clinically reported genomic variants for each patient derived by paired Whole Genome Sequencing.

Case ID	Somatic/ Germline	Gene	GRCh38 coordinates	Change
1	Somatic	*FBXW7*	4:152326137	c.1513C>T p.Arg505Cys
2	Somatic	*NA::PDGFD*	10:42342455::11:104074268	Translocation
2	Somatic	*PDGFD*	11:85358432-104075784	LOSS(1)
2	Somatic	*CCNY::ERC1*	10:35545945::12:1481332	Translocation
2	Somatic	*TNKS2::ERC1*	10:91846045::12:1483141	Translocation
2	Somatic	*DLG2::ERC1*	11:85358705::12:1483518	Translocation
2	Somatic	*PIK3C2G*	12:17514747-21246206	LOSS(0)
3	Mosaic variant	*TRIM28*	19:58548041 CTG>C	c.963_964delTG p.Glu322GlyfsTer34
3	Somatic	*TRIM28*	19:54792113-58586707	LOH(2)
4	Somatic	*AMER1*	X:64192215 G>A	c.1072C>T p.Arg358Ter
4	Somatic	*CREBBP*	16:3729745 G>GT	c.5302dupA p.Arg1768ThrfsTer198
4	Somatic	*AMER1*	X:63975071-65261038	LOSS(0)
4	Germline	*DIS3L2*	2:232146755-232176364	Exon 9 deletion
5	Somatic	*NSD2*	4:1961074 G>A	c.4395G>A p.Glu1099Lys
5	Somatic	*REST*	4:56910978 T>TTGGAACTCAGCGTCGTAGAACC	c.342_363dup22 p.Gln122GlyfsTer12
5	Germline	*REST*	4:53144872-57711698	LOSS(1)
5	Somatic	*NA*	11:196821-50820934	LOH(2)

Supplementary Table 2 – Case 2 somatic RNA fusion panel result (derived from Illumina TruSight panel)

Gene Fusion	ERC1 transcript	ERC1 Exon No.	ERC1 Coordinate	CCNY transcript	CCNY Exon No.	CCNY Coordinate
ERC1-CCNY	NM_178040.4	18	r.?_3123	NM_145012.6	8	r.580_?

Supplementary Figure 1 – case 3 radiology and histology

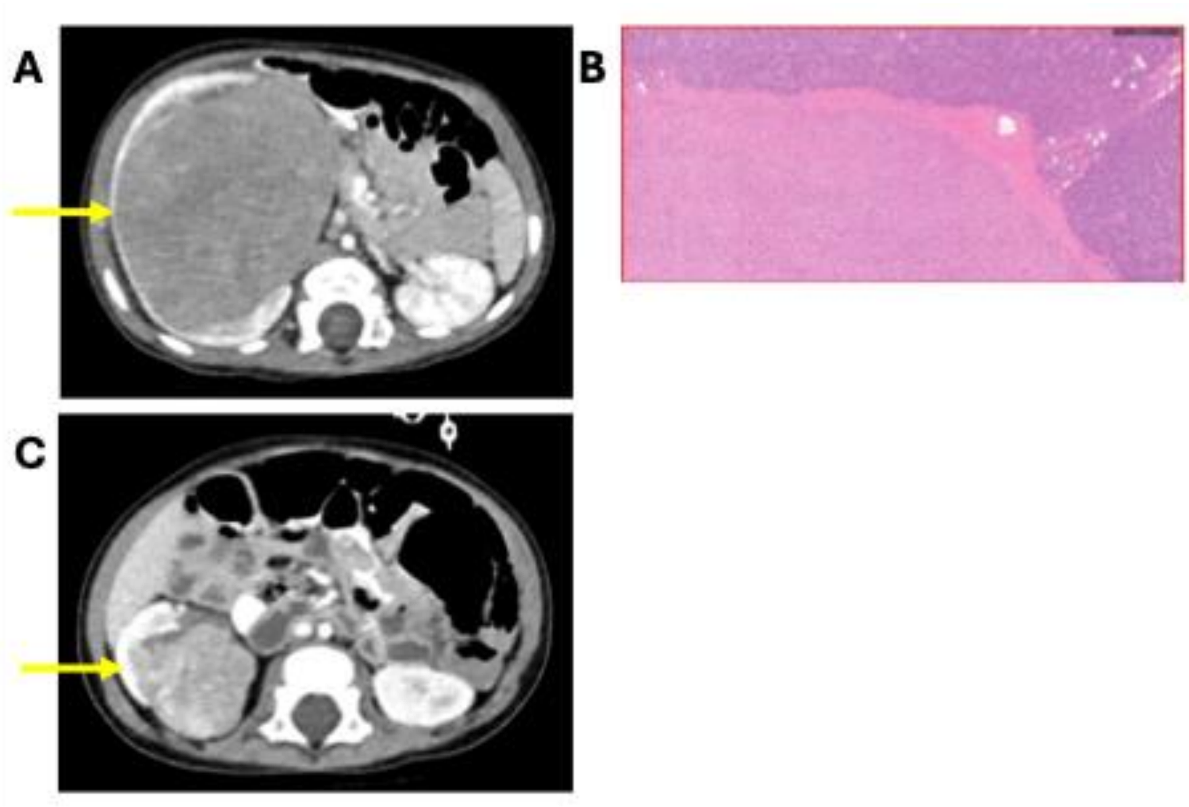

A) Axial slice from a nephrographic phase CT abdomen/pelvis performed at presentation showing a large heterogenous right renal tumour with presence of a characteristic claw sign (arrow). B) Tumour (epithelial WT) and normal kidney, including a clear fibrous pseudocapsule. C) Axial slice from a portal venous phase CT abdomen/pelvis performed following four weeks of pre-operative chemotherapy, showing a substantial interval reduction in the size of the right renal tumour (arrow)

Supplementary Figure 2 – case 4 radiology and histology

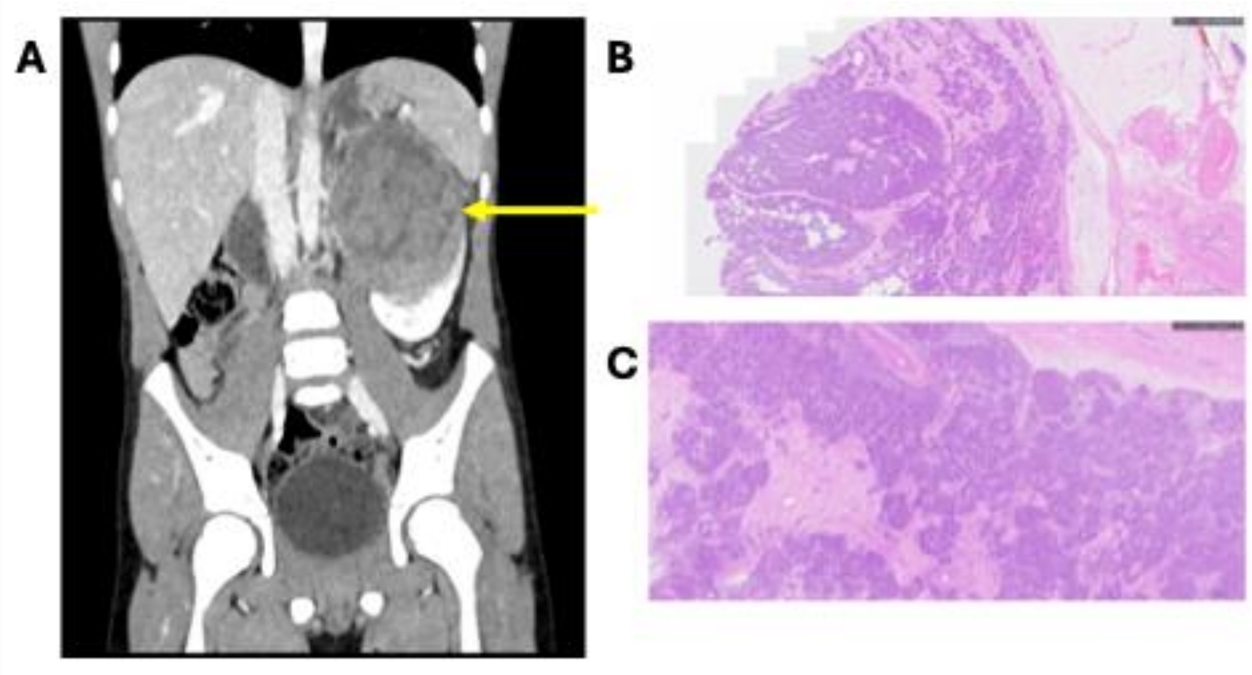

A) Coronal reformat from a corticomedullary phase CT abdomen/pelvis performed at presentation showing a large heterogenous left renal mass with evidence of a renal claw sign (arrow). The left adrenal gland was well visualized and separate from the mass, but there was a relatively large volume of hyperattenuating

suprarenal fluid, concerning for tumoral haemorrhage and rupture B-C) H&E resection specimens showing classical triphasic WT including blastema, stromal and epithelial components

Supplementary Figure 3 – case 5 radiology and histology

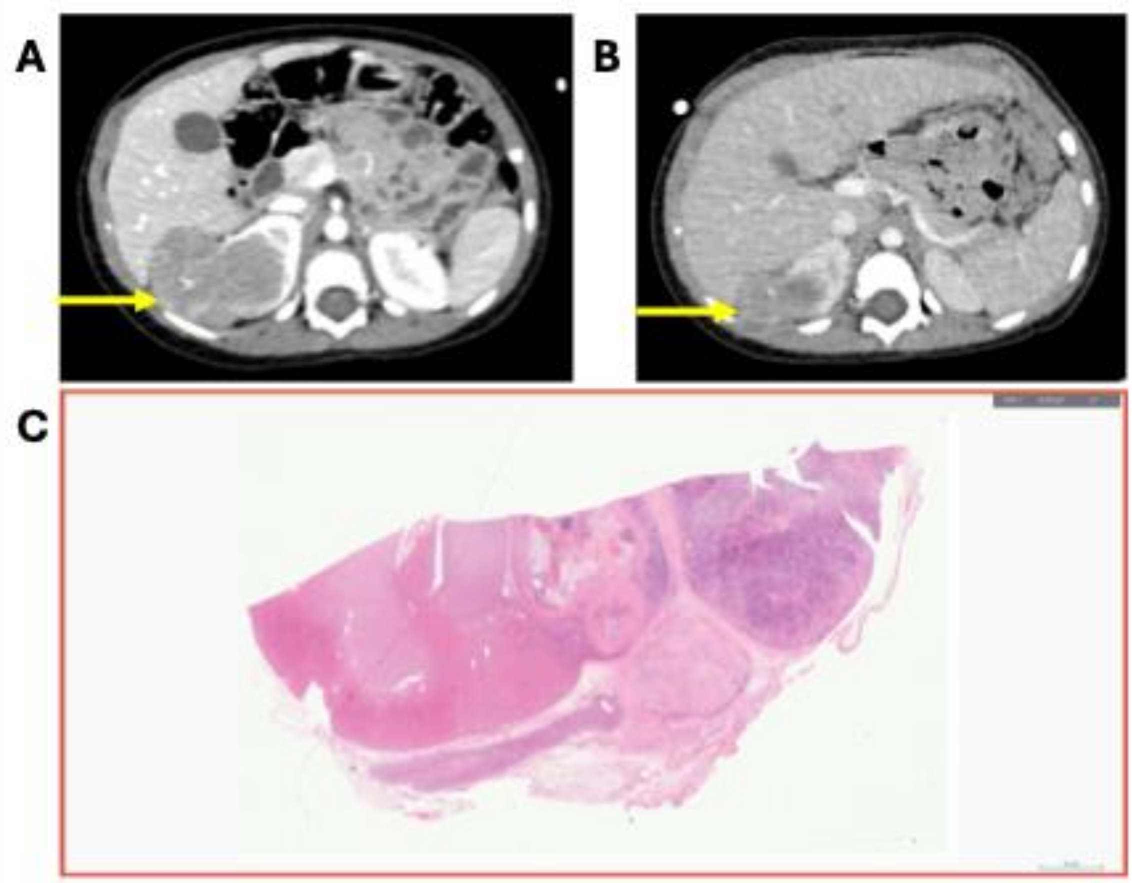

A) Axial slice from a portal corticomedullary phase CT of the abdomen and pelvis performed at presentation showing a bilobed mass arising from the upper pole of the right kidney with claw sign present (arrow). There was loss of the normal fat plane with the liver without definitive evidence of invasion. The lesion was separate from the right adrenal gland (not pictured and difficult to demonstrate on a single slice). B) Axial slice from a corticomedullary phase CT of the abdomen and pelvis performed following four weeks of pre-operative chemotherapy showing a substantial interval reduction in the size of the bilobed right renal tumour (arrow). There was persistent loss of the normal fat plane with the liver despite a reduction in the size of the primary tumour. C) Section of tumour and normal kidney interface, in which the classical triphasic WT appearance can be appreciated despite treatment induced change. This section shows formation of a fibrous pseudocapsule, which may be secondary to treatment effect. It may also reflect origin of this tumour from a nephrogenic rest. Elsewhere this pseudocapsule is absent, and the tumour is seen to merge into normal renal parenchyma
